# Development of PZN-PMN-PZT Piezoelectric Ceramics with High d_33_ and Q_m_ Values

**DOI:** 10.3390/ma15207070

**Published:** 2022-10-11

**Authors:** So-Won Kim, Hee-Chul Lee

**Affiliations:** Department of Advanced Materials Engineering, Tech University of Korea, Siheung 15073, Korea

**Keywords:** piezoelectricity, relaxor, doping materials, Zr/Ti ratio, piezoelectric properties

## Abstract

To achieve good long-term temperature stability in devices used in energy-conversion applications, this study is aimed at developing combined ceramics, referred to as PZN-PMN-PZT, comprising Pb(Zn_1/3_Nb_2/3_)O_3_ (PZN) and Pb(Mn_1/3_Nb_2/3_)O_3_ (PMN), which are typical relaxor ferroelectric materials, and Pb(Zr_,_Ti)O_3_ (PZT). The piezoelectric properties were compared based on several parameters according to the change in the composition ratio between relaxor materials, amounts of Sb_2_O_3_ dopant, and Zr/Ti ratio in the PZT system. Finally, we established optimal poling conditions to improve the electrical properties of the optimized piezoelectric material, based on the evaluation of ceramic properties according to the applied voltage during the poling process. The optimized composition of the investigated piezoelectric ceramics is represented by 0.14PZN-0.06PMN-0.80PbZr_0.49_Ti_0.51_ + 0.3 wt.% CuO + 0.3 wt.% Fe_2_O_3_ with 0.1 wt.% Sb_2_O_3_ doping, which yielded the superior properties (d_33_ = 361 pC/N, Q_m_ = 1234, T_c_ = 306 °C).

## 1. Introduction

Piezoelectric ceramics, which interconvert electrical and mechanical signals, have been widely integrated in devices such as actuators, sensors, ultrasonic transducers, and transformers. Because of the constant demand for the miniaturization and thinning of electronic products, the application of piezoelectric ceramics is broadly expanding to mobile devices, medical devices, and energy harvesters [1,2,3,4]. The benefits of energy conversion devices, such as piezoelectric transformers, which possess the ability to convert electrical energy into mechanical energy and reconvert it into electrical energy, include a simple design, high efficiency, compact form, and light weight. Because a piezoelectric device simultaneously utilizes the reverse and direct piezoelectric effects, it is necessary to develop outstanding piezoelectric materials that can simultaneously yield a high piezoelectric coefficient (d_33_) and electromechanical coupling factor (k_p_) to enhance the energy conversion efficiency, as well as a high mechanical quality factor (Q_m_) to reduce heat loss [5,6,7,8].

Since the discovery of PZT, many researchers have reported a three-component system including relaxor ferroelectric materials based on PZT, such as Pb(Zn_1/3_Nb_2/3_)O_3_–Pb(Zr,Ti)O_3_ (PZN-PZT) [9], Pb(Mn_1/3_Nb_2/3_)O_3_–Pb(Zr,Ti)O_3_ (PMN-PZT) [10], Pb(Fe_1/2_Nb_1/2_) O_3_–Pb(Zr,Ti)O_3_ (PFN-PZT) [11], and Pb(Sb_1/2_Nb_1/2_)O_3_–Pb(Zr,Ti)O_3_ (PSN-PZT) [12], allowing the piezoelectric characteristics to be changed in various ways based on the material’s purpose. However, these three-component piezoelectric ceramic compositions generally have low dielectric constant and mechanical quality factor, which limits their applications to energy conversion devices, so research on compositions, such as four-component systems, have been actively conducted. 

In general, there are many previous reports of an improvement in the properties by changing the molar ratio of PZT to compensate for the deterioration of properties caused by the relaxor ferroelectrics employed in PZT. However, this study intends to analyze the effects of the ratio between relaxor ferroelectrics constituting a four-component system on the properties.

In a previous study, Pb(Ni_1/3_Nb_2/3_)O_3_ (PNN) and Pb(Mn_1/3_Nb_2/3_)O_3_ (PMN), which are typical relaxor ferroelectric materials, were employed in Pb(Zr,Ti)O_3_ (PZT) piezoelectric ceramics. It was confirmed that the PNN-PMN-PZT ceramic exhibited a high dielectric constant and piezoelectric properties [13]. The devices used in energy conversion application should possess excellent temperature stability even after long-term use. Thus, this study aimed to develop a material with the novel composition of PZN-PMN-PZT that exhibits better temperature stability by employing PZN (Pb(Zn_1/3_Nb_2/3_)O_3_) (T_c_~140 °C) with a higher phase transition temperature than that of PNN (T_c_~120 °C) and adjusting the Zr/Ti composition ratio in the PZT system to compensate for the low phase transition temperature of the PNN-PMN-PZT ceramic [14,15,16,17].

Thus, to achieve the objectives of the study, the PZT molar ratio was fixed, and the piezoelectric properties were compared and analyzed according to the change in the composition ratio between PZN and PMN, which are relaxor materials using the four-component system PZN-PMN-PZT-based ceramic. To reduce the sintering temperature of the basic composition, 0.3 wt.% each of CuO and Fe_2_O_3_ were added as sintering aids, and Sb^3+^, which serves as a hardener, was also added as a dopant after synthesizing the four-component material to study the doping effect in the novel composition. In addition, the effect of crystal structure difference according to the Zr/Ti ratio on the piezoelectric properties and temperature stability of the ceramic was studied. Finally, to improve the electrical properties of the optimized piezoelectric material, the optimal poling condition was established based on the evaluation of ceramic properties according to the applied voltage during the poling process.

## 2. Materials and Methods

**Figure 1:**
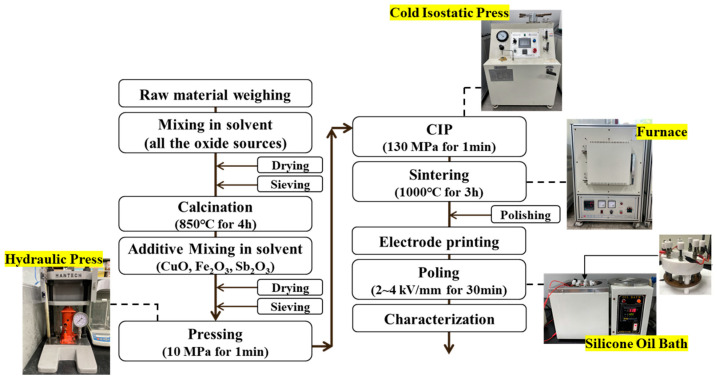
Process flow diagram describing fabrication of PZN-PMN-PZT ceramics.

The composition formula (0.20−x)Pb(Zn_1/3_Nb_2/3_)O_3_-xPb(Mn_1/3_Nb_2/3_)O_3_-0.80Pb(Zr_1−y_Ti_y_)O_3_ (PZN-PMN-PZT, x = 0.02~0.18, y = 0.5~0.54) + 0.3 wt.% CuO + 0.3 wt.% Fe_2_O_3_ was used in this study. Figure 1 shows the process flow chart for the fabrication of the PZN-PMN-PZT ceramics. The weight of the raw material powder depended on the composition ratio, and it was mixed in an ethanol solvent for 24 h using a ball mill and then dried to obtain a uniform powder. Subsequently, calcination was carried out at a constant temperature of 850 °C for 4 h, and a certain amount of Sb_2_O_3_ dopant in the range 0.1–0.7 wt.% was added and mixed for an additional 24 h. An appropriate volume of 10 wt.% poly vinyl alcohol (PVA) aqueous solution was added to the powder as a binder, and a pressure of approximately 10 MPa was applied to form a 20 mm diameter disk. The molded specimen was subjected to a water pressure of 130 MPa for 1 min using a cold isostatic pressing (CIP) process. The sintering was performed at 500 °C for 1 h to remove the binder and then at 1000 °C for 3 h. The degree of densification was confirmed by observing the microstructure of the sintered specimen using a scanning electron microscope (Nova Navo SEM 450, ThermoFisher, Waltham, MA, USA), and the crystallographic structure was analyzed at a diffraction angle from 20 to 60° using an X-ray diffraction analyzer (XRD, BRUKER, Billerica, MA, USA).

To study the properties of the sintered specimen, both sides were polished to a thickness of 2 mm, coated with Ag electrode, and then annealed at 600 °C for 30 min. The electrode specimen was poled in a silicone oil bath at 120 °C by applying an electric field in the range of 2–4 kV/mm for 30 min, followed by aging for 24 h in air. For the poled specimen, the piezoelectric coefficient (d_33_) was measured using a d_33_ meter (Model YE2730, HANTECH, Gunpo, Korea), while the electromechanical coupling factor (k_p_) and the mechanical quality factor (Q_m_) were calculated according to the IEEE standard method using an impedance analyzer (Model 4990A, Keysight Technologies Inc., Santa Rosa, CA, USA). To determine the Curie temperature (T_c_), the specimen was placed in a chamber system (Poly K Technologies, Philipsburg, PA, USA), and the change in dielectric constant as a function of temperature in the range of 20–500 °C was measured at different capacitance values under 1 kHz by connecting it to an LCR meter (E4980, Agilent, Santa Clara, CA, USA).

## 3. Experimental Results and Discussion

**Figure 2:**
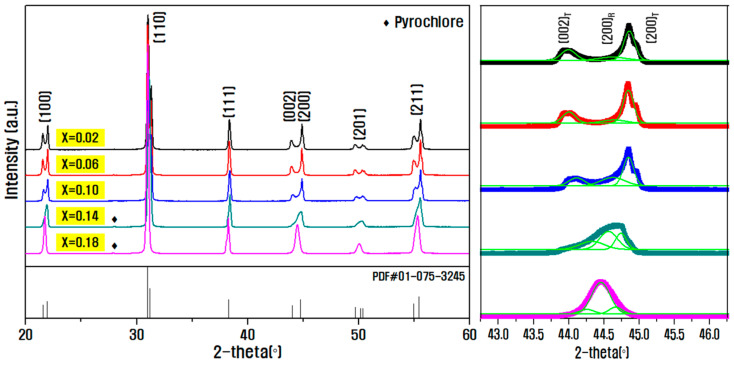
X-ray diffraction pattern analysis results according to PMN content in the (0.20−x)PZN-xPMN-0.80Pb(Zr_0.5_Ti_0.5_)O_3_ ceramic.

Figure 2 shows the X-ray diffraction pattern of the sintered ceramic according to the composition ratio of PZN and PMN in the (0.20−x)PZN-xPMN-0.80Pb(Zr_0.5_Ti_0.5_)O_3_ (x = 0.02–0.18) ceramic. From the diffraction pattern analysis results, it can be seen that stable perovskite structures without secondary phases were formed in all composition ranges of ceramic. The enlargement of the diffraction pattern in the 2-θ range 44–46° shows the formation of (200)_R_ rhombohedral diffraction peak between the tetragonal (002)_T_ and (200)_T_ peaks with increasing molar ratio of PMN. This phenomenon occurred mainly because Mn^4+^ ions were partially reduced into Mn^2+^ (0.82 Å) or Mn^3+^ (0.65 Å) during the sintering process substituting the Zr^4+^ (0.72 Å) or Ti^4+^ (0.61 Å) position at the B-site in the perovskite structure. The lattice was distorted because of the large radius of the Mn ion, and as the movement of ions became more difficult because of the stuffing effect, the phase transition from rhombohedral to tetragonal slowed down, thus stabilizing the rhombohedral phase [18,19,20].

**Figure 3:**
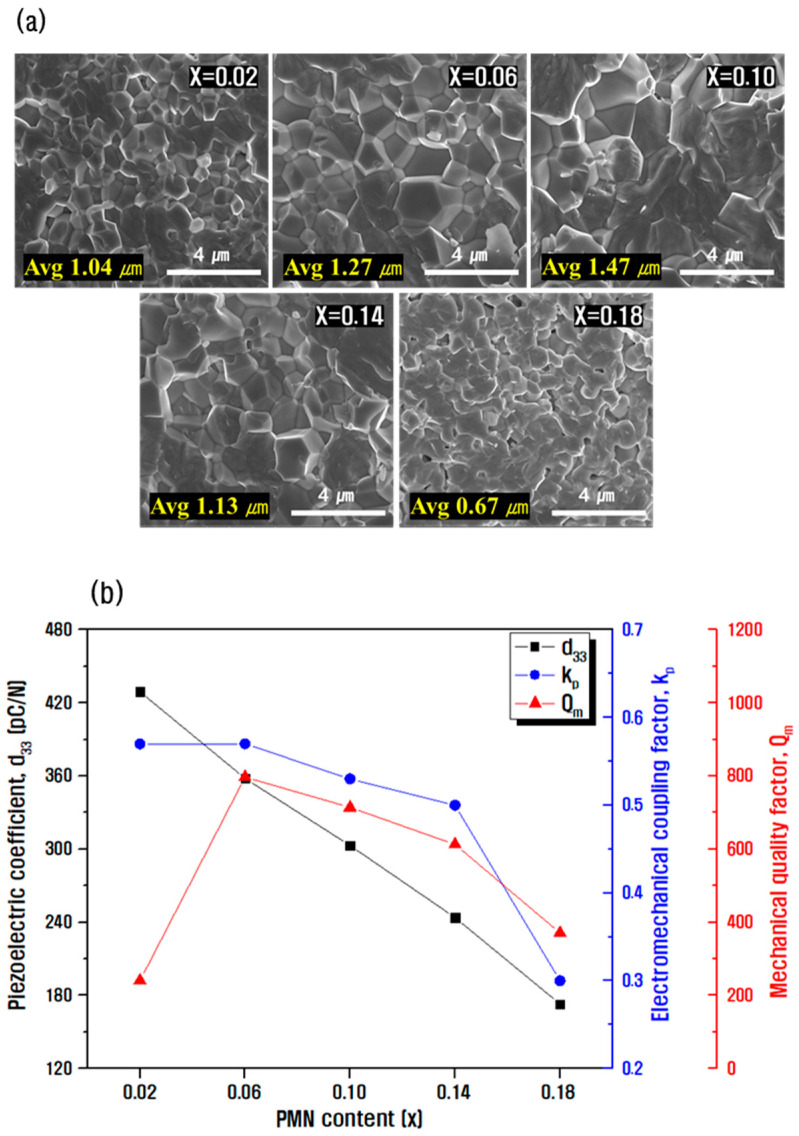
(**a**) FE-SEM image of fracture surfaces (Avg: the average of grain size) and (**b**) change in electrical properties represented by d_33_, k_p_, and Q_m_ as a function of PMN content in (0.20−x)PZN-xPMN-0.80Pb(Zr_0.5_Ti_0.5_)O_3_ ceramic.

To determine the tetragonality of the crystal structure according to the composition ratio of PZN and PMN, the lattice parameters a and c were determined using the value of (200) and (002) plane in the 2-θ range of 44–46° in the X-ray diffraction pattern. Further, tetragonality (T = c/a) and theoretical density were calculated, while the actual density was measured using the Archimedes’ method, as shown in Table 1 [21]. Because the Mn ion with a larger ionic radius occupied the B-site as the PMN content increased, the lattice constant increased and the anisotropic tetragonal phase decreased, which resulted in a decrease in the tetragonality of the ceramic to 1.019–1. In general, as the lattice volume increases, the XRD peak shifts to a lower angle, but no tendency could be observed for the 2-theta angle as the PMN content increased, as shown in Figure 2. In addition, the relative density was calculated based on the ratio of the actual density to the theoretical density of each composition. Here, the density value decreased as the PMN content increased, and the maximum relative density decreased from 99% to 92%. The density of the ceramic may be correlated to the microstructure and porosity, as seen in the SEM image in Figure 3a.

The SEM image in Figure 3a shows the microstructure of fracture surfaces of the sintered ceramic according to the composition ratio of PZN and PMN, while the average particle size was calculated using linear intercept analysis. As the anisotropy of crystal structure decreased because of the PMN substitution, the crystal grains grew, resulting in a maximum value of the average particle size at x = 0.10 of 1.47 μm. For x ≥ 0.14, the pores increased, and the growth of grains was inhibited because of the distribution of the impurities that could not be dissolved beyond the solubility limit in the grain boundary layer [22,23,24]. Figure 3b shows the measurement results of electrical properties according to the composition ratio. As the PMN content increased, the piezoelectric coefficient and electromechanical coupling coefficient decreased. On the other hand, the mechanical quality factor increased until x = 0.06, and its value decreased thereafter. This might occur because the space charge increased as the oxygen ion vacancies located at the face center of the perovskite lattice were created to maintain electrical neutrality during the Mn ion substitution for B-site in the perovskite lattice. This further resulted in an increase in the internal electric field within the particle, and the domain motion was suppressed by this electric field, resulting in a decrease in polarization efficiency. The decrease in the mechanical quality factor at x ≥ 0.10 may be attributed to the rapid decrease in density [20,25,26,27].

The doping effect was studied to develop a novel composition by adding a hardener, Sb_2_O_3_, as a dopant to improve the mechanical quality factor to the basic composition at x = 0.06. It exhibited excellent piezoelectric properties with d_33_ = 358 pC/N, k_p_ = 0.57, and Q_m_ = 797 based on the experimental results according to the composition ratio of relaxors in PZN-PMN-PZT ceramics.

**Figure 4:**
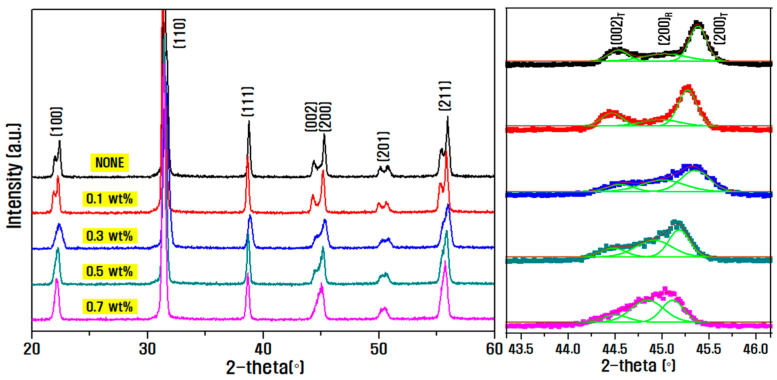
X-ray diffraction pattern analysis results according to the Sb_2_O_3_ doping amount in PZN-PMN-PZT ceramic.

Figure 4 shows the X-ray diffraction patterns according to the amount of Sb_2_O_3_ dopant in the basic composition of 0.14PZN-0.06PMN-0.8PZT. It can be seen that the (200)_R_ diffraction peak between 44 and 46° gradually increased with the Sb_2_O_3_ dopant content. This might be because the Sb^3+^(0.76 Å) ion with a similar radius occupied the high-valence Zr^4+^(0.72 Å) ion site, thus stabilizing the isotropic rhombohedral phase [28].

**Figure 5:**
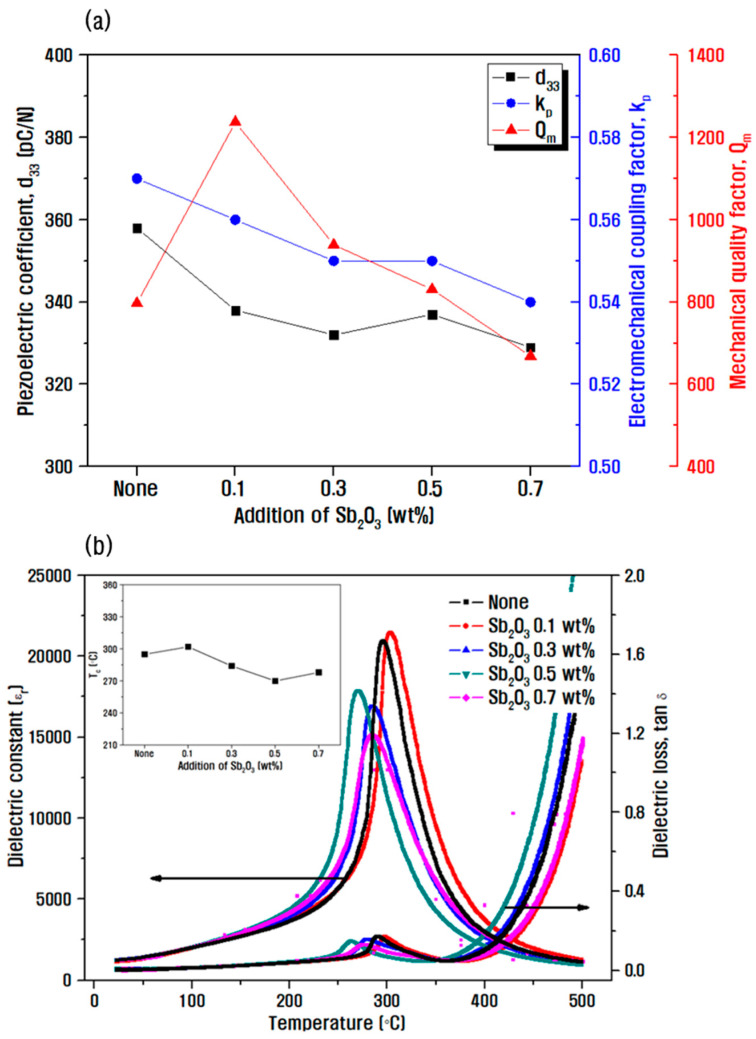
(**a**) change in electrical properties represented by d_33_, k_p_, and Q_m_ according to Sb_2_O_3_ doping amount, and (**b**) dielectric properties according to temperature change at 1 kHz measurement (inset graph: the change in Curie temperature according to Sb_2_O_3_ amount) in PZN-PMN-PZT ceramic.

Figure 5 shows the results of the study of electrical properties according to the amount of Sb_2_O_3_ dopant. As the amount of Sb_2_O_3_ increased, the piezoelectric coefficient and the electromechanical coupling coefficient decreased, and on the contrary, the mechanical quality factor tended to increase in Figure 5a. The decrease in poling efficiency may be attributed to the decrease in domain mobility when the Sb^3+^ ion replaced the high-valence Zr^4+^ position at B-site, similar to the Mn ion substitution effect described in Figure 3b [29,30]. The mechanical quality factor exhibited a maximum value of 1237 for Sb_2_O_3_ 0.1 wt.%, and it gradually decreased as the added amount increased over 0.3 wt.%. The increase in the amount of additive resulted in more defects, such as oxygen vacancies, in the sintered body, and deterioration in the density and the mechanical quality factor [27]. Figure 5b shows dielectric properties according to the temperature change at 1 kHz measurement. The maximum Curie temperature, which is the temperature at the maximum permittivity value, was 302 °C at 0.1 wt.% Sb_2_O_3._ However, the further addition of Sb_2_O_3_ resulted in the decline of curie temperature and this phenomenon may be related to the lattice anisotropy decrease with the increase of Sb_2_O_3_ content [30].

In the phase transition region, the movement of the interphase boundary will induce energy loss abruptly, leading to the loss peak around curie temperature. There is a sudden increase of the loss peak, with a peak position together with the T_c_. This may be associated with the displacement of domain walls near the phase transition temperature. The dielectric loss then begins to increase again at the temperatures over T_c_.

The composition of 0.14PZN-0.06PMN-0.80PbZr_0.5_Ti_0.5_ 0.1 wt.% Sb_2_O_3_ with piezoelectric properties d_33_ = 338 pC/N, k_p_ = 0.56, and Q_m_ = 1237 in Figure 5 was considered suitable for energy conversion devices. However, the phase transition temperature is as low as 302 °C in this composition, so an additional experiment was conducted to achieve a piezoelectric ceramic composition with better temperature stability by varying the Zr/Ti ratio in PZT.

**Figure 6:**
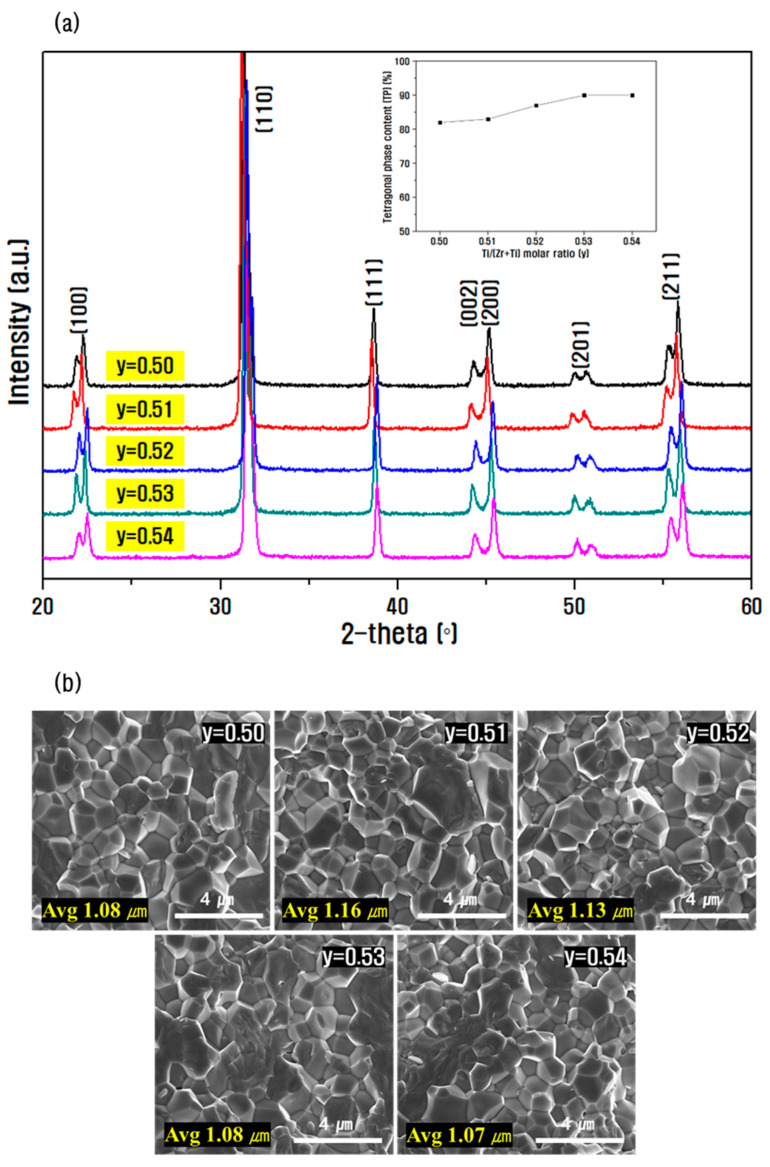
(**a**) X-ray diffraction pattern (inset graph: content change of tetragonal phase) and (**b**) FE-SEM image of fracture surfaces of ceramic according to Zr/Ti ratio in 0.14PZN-0.06PMN-0.80PbZr_1−y_Ti_y_·0.1 wt.% Sb_2_O_3_ composition.

Figure 6 shows the X-ray diffraction pattern and SEM image of the fracture surfaces according to the Zr/Ti composition change (y = 0.5–0.54) in the 0.14PZN-0.06PMN-0.80PbZr_1−y_Ti_y_·0.1 wt.% Sb_2_O_3_ ceramic. No secondary phase was seen in the diffraction pattern of all compositions in Figure 6a. As the Zr/Ti ratio decreased, the peaks of tetragonal (002), (200), and rhombohedral (200) on the diffraction pattern in the 2-θ range 44–46° were clearly split, confirming the phase transition from rhombohedral to tetragonal. The tetragonal phase fraction calculated based on the comparison of the relative intensity ratios of the splitting peaks indicated an increase from 82% at the Zr/Ti ratio 50/50 to 90% at the 46/54 composition, and this may be attributed to the tetragonal PbTiO_3_. In general, the MPB (morphotropic phase boundary) region of pure PZT is near the Zr/Ti ratio 53/47, while in this composition, the lattice constant was altered and the MPB region was slightly shifted toward Ti-rich because of the substitution of Zn^2+^(0.74 Å), Mn^2+^(0.82 Å), Nb^5+^(0.64 Å), and Sb^3+^(0.76 Å) elements for the B-site of the perovskite structure [31,32,33]. From Figure 6b, it can be confirmed that all compositions possessed a dense structure without pores, and the particle size increased from approximately 1.08 μm at the Zr/Ti ratio 50/50 to 1.16 μm at the 49/51 ratio. Further, as the Zr/Ti ratio decreased, the particle size was slightly reduced because of the increase in the anisotropy of the crystal lattice and the stress inside the lattice caused by the enhancement of tetragonal phase, thus inhibiting the growth of crystal grains [34]. However, there was no significant difference according to the Zr/Ti composition ratio.

**Figure 7:**
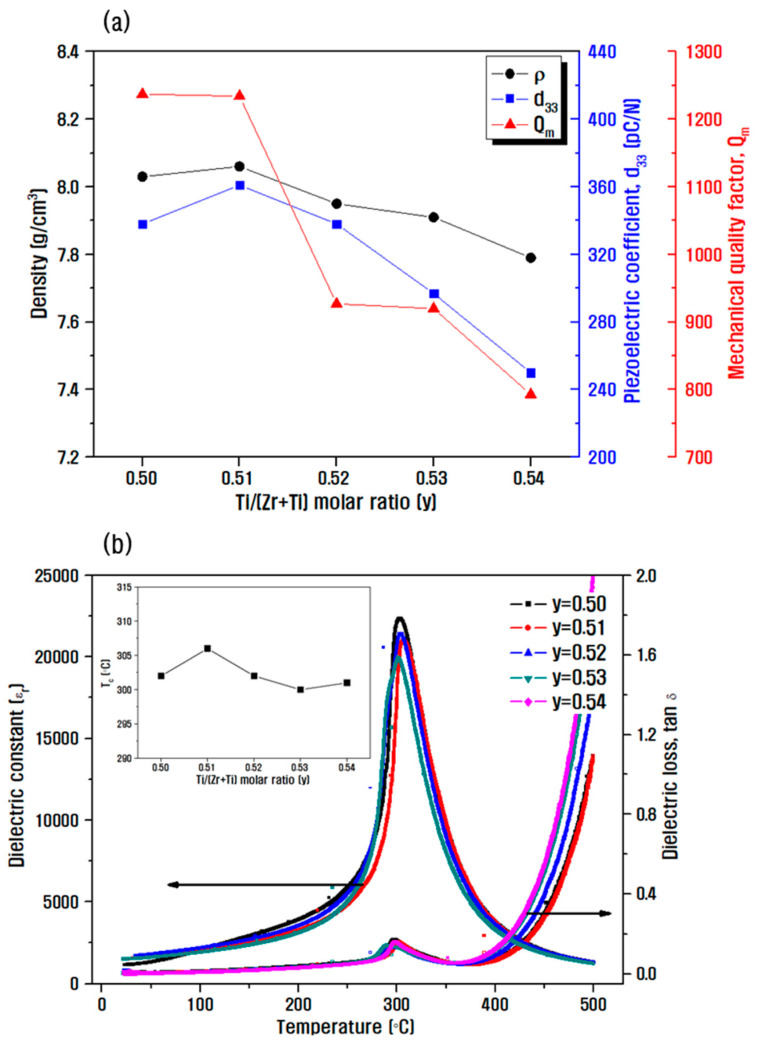
(**a**) Change in density and electrical properties represented by d_33_ and Q_m_, (**b**) dielectric properties with respect to temperature change at 1 kHz measurement (inset graph: Curie temperature change) according to the Zr/Ti ratio change in the basic composition of 0.14PZN-0.06PMN-0.80PbZ_1−y_T_y_ and composition added with 0.1 wt.% Sb_2_O_3_.

Figure 7 shows the results of the density and electrical properties measurement according to the Zr/Ti composition change. In Figure 7a, the maximum density under a decreasing Zr/Ti ratio is 8.06 g/cm^3^ and relative density is around 99% at the composition ratio of 49/51. Thus, the density value decreased slightly as the particle size decreased. In addition, as the composition of PbTiO_3_ with high-anisotropy crystal lattice increased, it approached the morphotropic phase boundary region where the tetragonal phase with six polarizable axes and the rhombohedral phase with eight polarizable axes coexisted. Further, the poling efficiency increased, resulting in a maximum value of piezoelectric properties at the Zr/Ti ratio 49/51 with d_33_ and Q_m_ of 361 pC/N and 1234, respectively. When it migrated from the morphotropic phase boundary region, the transition to tetragonal structure gradually occurred, and the piezoelectric properties were degraded from the composition 48/52, as shown in Figure 6a [35,36]. In Figure 7b, the Curie temperature was determined by measuring the change in the dielectric constant with temperature variations. In general, PbTiO_3_ (T_c_~490 °C) has a higher Curie temperature than that of PbZrO_3_ (T_c_~230 °C), and thus a higher value of 306 °C was confirmed at the Ti-rich 49/51 composition than that at the Zr/Ti ratio 50/50 [24,37,38]. However, the Curie temperature decreased in the Zr/Ti ratio range from 48/52 to 46/54, and the trend of decrease was similar to that of the piezoelectric properties. As a result, by changing the Zr/Ti ratio in the optimized 0.14PZN-0.06PMN-0.80PbZr_1−y_Ti_y_ basic composition and the composition with added 0.1 wt.% Sb_2_O_3_, a piezoelectric material with a novel composition and a high Curie temperature of 306 °C and without significant deterioration of d_33_ and Q_m_ compared to those of the existing composition was developed.

**Figure 8:**
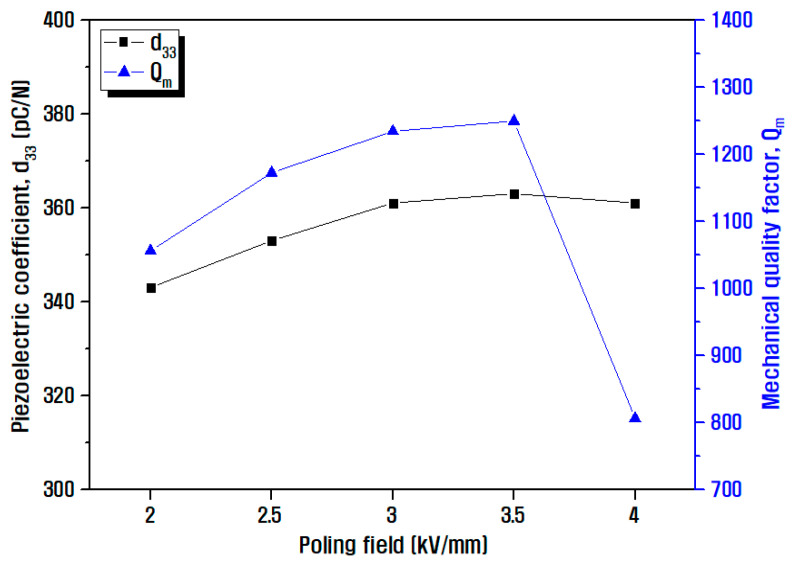
Electrical properties measurement results of the ceramic represented by d_33_ and Q_m_ with respect to the poling field.

Figure 8 shows the electrical properties of the ceramics that were evaluated by varying poling field on a specimen prepared using the optimized composition in this study. Previously, all the optimized materials were poled under the same electric field of 3 kV/mm. However, to examine the degree of improvement in electrical properties at different applied voltage controlled in the poling process, the properties of the ceramic were investigated according to the poling field. In general, the degree of domain orientation and the energy conversion efficiency improved with the increase in the external energy such as poling field, and similarly, the poling efficiency increased for a maximum value of d_33_ = 363 pC/N and Q_m_ = 1249 at the poling field of 3.5 kV/mm. When the electric field increased further, the piezoelectric properties value decreased because the charge carriers inside the specimen increased when the specimen was poled with the excessively high electric field, thereby resulting in a decrease in the insulation resistance and an increase in the internal current of the ceramic. Consequently, the poling field applied to the crystal decreased, resulting in a subsequent decrease in the poling efficiency. Therefore, it could be confirmed that the selection of appropriate poling conditions is essential even for the same material because the internal electrical properties may vary depending on the poling conditions that are integrated [39,40].

## 4. Conclusions

The Pb(Zn_1/3_Nb_2/3_)O_3_-Pb(Mn_1/3_Nb_2/3_)O_3_-Pb(Zr,Ti)O_3_ composition was studied according to the ratio of PZN and PMN and the added Sb_2_O_3_ with the aim of developing a piezoelectric material with a novel composition that satisfies a high d_33_ to enhance the energy conversion efficiency as well as a high Q_m_ and T_c_ to reduce heat loss for energy conversion devices. The substitution of PMN, which is a hard relaxor, in the three-component system of PZN-PZT resulted in a gradual decrease in the tetragonality. Further, the rhombohedral phase was stabilized as the PMN content increased, while in the case of 6 mol% PMN substitution, the relative density was approximately 98% and the value of mechanical quality factor was maximum. The addition of the Sb^3+^ element, which acts as a hardener, for use as a dopant resulted in the formation of oxygen vacancies in the perovskite crystal. This further created the acceptor substitution effect. In the case of 0.1 wt.% addition, the d_33_ and Q_m_ exhibited values of 338 pC/N and 1237, which were relatively high compared to those in the case without additive. In addition, the composition with Zr/Ti ratio 49/51 maintained outstanding piezoelectric properties with d_33_ = 361 pC/N and Q_m_ = 1234 without significant deterioration in properties compared to the basic composition. The phase transition temperature was found to be 306 °C, which confirmed that a material with an improved temperature stability was developed. The piezoelectric material with a novel composition developed in this study not only has a high d_33_ and Q_m_, which results in low heat dissipation during high-power operations, but also a high phase transition temperature, confirming that the composition can be potentially integrated in materials aimed at manufacturing energy conversion devices.

## Figures and Tables

**Table 1:** Lattice constants, tetragonality and density measurement results according to PMN content in (0.20−x)PZN-xPMN-0.80Pb(Zr_0.5_Ti_0.5_)O_3_ ceramic.

PMN Content (x)	Lattice Parameters		Tetragonality (c/a)	Density (g/cm^3^)		Relative Density (%)
	a(Å)	c(Å)		Theoretical	Experimental	
0.02	4.038	4.118	1.019	8.10	8.02	99.0
0.06	4.040	4.118	1.019	8.09	7.96	98.4
0.10	4.038	4.114	1.018	8.11	7.78	95.9
0.14	4.048	4.094	1.011	8.11	7.68	94.7
0.18	4.088	4.092	1.000	7.96	7.32	92.0

## Data Availability

The data presented in this study are contained within the article.
